# Complete chloroplast genome of *Oncidium *Gower Ramsey and evaluation of molecular markers for identification and breeding in Oncidiinae

**DOI:** 10.1186/1471-2229-10-68

**Published:** 2010-04-16

**Authors:** Fu-Hui Wu, Ming-Tsair Chan, De-Chih Liao, Chen-Tran Hsu, Yi-Wei Lee, Henry Daniell, Melvin R Duvall, Choun-Sea Lin

**Affiliations:** 1 Agricultural Biotechnology Research Center, Academia Sinica, Taipei, Taiwan; 2 Department of Molecular Biology and Microbiology, College of Medicine, Biomolecular Science Building, University of Central Florida, Orlando, USA; 3 Department of Biology, Northern Illinois University, DeKalb, USA

## Abstract

**Background:**

*Oncidium *spp. produce commercially important orchid cut flowers. However, they are amenable to intergeneric and inter-specific crossing making phylogenetic identification very difficult. Molecular markers derived from the chloroplast genome can provide useful tools for phylogenetic resolution.

**Results:**

The complete chloroplast genome of the economically important *Oncidium *variety *Onc*. Gower Ramsey (Accession no. GQ324949) was determined using a polymerase chain reaction (PCR) and Sanger based ABI sequencing. The length of the *Oncidium *chloroplast genome is 146,484 bp. Genome structure, gene order and orientation are similar to *Phalaenopsis*, but differ from typical Poaceae, other monocots for which there are several published chloroplast (cp) genome. The *Onc*. Gower Ramsey chloroplast-encoded *NADH dehydrogenase *(*ndh*) genes, except *ndhE*, lack apparent functions. Deletion and other types of mutations were also found in the *ndh *genes of 15 other economically important Oncidiinae varieties, except *ndhE *in some species. The positions of some species in the evolution and taxonomy of Oncidiinae are difficult to identify. To identify the relationships between the 15 Oncidiinae hybrids, eight regions of the *Onc*. Gower Ramsey chloroplast genome were amplified by PCR for phylogenetic analysis. A total of 7042 bp derived from the eight regions could identify the relationships at the species level, which were supported by high bootstrap values. One particular 1846 bp region, derived from two PCR products (*trnH*^GUG ^-*psbA *and *trnF*^GAA^-*ndhJ*) was adequate for correct phylogenetic placement of 13 of the 15 varieties (with the exception of *Degarmoara *Flying High and *Odontoglossum *Violetta von Holm). Thus the chloroplast genome provides a useful molecular marker for species identifications.

**Conclusion:**

In this report, we used *Phalaenopsis. aphrodite *as a prototype for primer design to complete the *Onc*. Gower Ramsey genome sequence. Gene annotation showed that most of the *ndh *genes inOncidiinae, with the exception of *ndhE*, are non-functional. This phenomenon was observed in all of the Oncidiinae species tested. The genes and chloroplast DNA regions that would be the most useful for phylogenetic analysis were determined to be the *trnH*^GUG^-*psbA *and the *trnF*^GAA^-*ndhJ *regions. We conclude that complete chloroplast genome information is useful for plant phylogenetic and evolutionary studies in *Oncidium *with applications for breeding and variety identification.

## Background

The Oncidiinae subtribe of the Orchidaceae family, consisting of about 70 closely related genera with over 1000 species, is divided into five alliances, with *Oncidium *as its largest genus [1]. From the perspective of cellular biology, ecology and morphology, *Oncidium *is the most diverse genus in the Orchidaceae. Traditionally, the taxonomy of the Oncidiinae tribe is based on the morphology of the flower [2]; however, morphology is affected by environmental factors, and over time flower morphologies have evolved convergently. The positions of some species in the evolution and taxonomy of Oncidiinae are therefore difficult to identify. Accurate identification is further complicated by the ease with which Oncidiinae can be crossed intergenerically, as indicated by the 107 intergeneric hybrids reported [1] and the fact that more than 2200 hybrids (about 20% in the *Oncidium *group) have been re-distributed into other genera.

Different molecular marker techniques such as terminal restriction fragment length polymorphism (TRFL), arbitrarily primed polymerase chain reaction (AP-PCR), DNA amplification fingerprinting (DAF), and random amplification polymorphism DNA (RAPD)] are available to conduct genetic analyses by PCR and provide information about evolution that is useful for taxonomy. Tsai *et al*. [3] used 257 RAPD markers to investigate the relationships between 24 species of Oncidiinae, and found that the species could be separated into seven groups; however, Tsai and colleagues were unable to identify the more detailed relationships among these species.

Although there are three different genomes in plants, chloroplast DNA (cpDNA) is in many respects the genome of choice for taxonomic studies in orchids [2] as well as other species [4,5]. There are many advantages to using cpDNA for taxonomy and evolutionary research: (1) the size of cpDNA is small, with high copy number and simple structure; (2) when compared to the mitochondrial and nuclear genome, cpDNA gene content and arrangement are more conserved, making it easier to design primers and clone genes; (3) cpDNA is maternally inherited and thus without the genetic reassortment that interferes with the molecular phylogenetic relationships [4,5].

The chloroplast genome is a circular chromosome of 120~220 kb that consists of two inverted repeats (IRa and IRb), a large single-copy region (LSC), and small single-copy region (SSC). This conserved structure and sequence information provides a resource for primer design for other cpDNA sequencing by PCR [6]. This approach has been used for the sequencing of two bamboo cpDNA genomes [7]. As chloroplast genome of one member of the Orchidaceae family, *Phalaenopsis aphrodite*, has already been published [8], it is very useful to sequence complete cpDNA from another orchid, such as *Oncidium *using PCR.

The Chloroplast genome also has applications in plant biotechnology. Chloroplast genetic engineering offers a number of unique advantages, including high levels of transgene expression, multi-gene engineering in a single transformation event, transgene containment via maternal inheritance and a lack of gene silencing and position effects [9,10]. However, the lack of complete chloroplast genome sequences is still a major limitation to extending this technology. Additional information about the chloroplast genome would, thus, be of great value in advancing orchid biotechnology.

In this study, we designed primers based on the *P. aphrodite *cpDNA and used them to identify the cpDNA of *Onc*. Gower Ramsey, an important cut flower orchid. Such primers were also used to investigate the *NADH dehydrogenase *(*ndh*) gene deletion patterns in 15 members of the Oncidiinae, and sequence amplified DNA regions to undertake phylogenetic analyses broadly across the angiosperms and at the species level.

## Methods

### Plant materials

**Figure 1:**
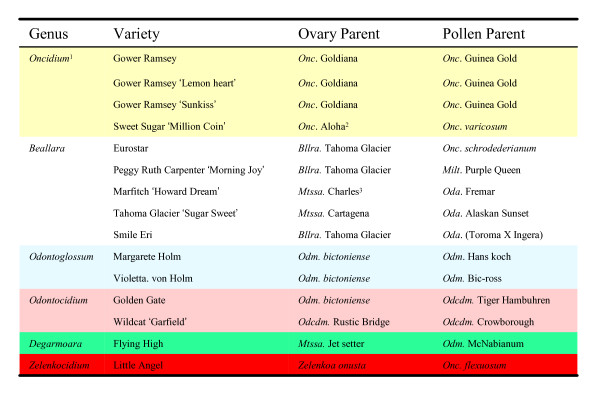
**Parents of 15 varieties of Oncidiinae**. ^1^Yellow background: *Oncidium*; white: *Beallara*; blue: *Odontoglossum*; purple: *Odontocidium*; green: *Degarmoara*; red: *Zelenkocidium*. ^2^*Onc*. Aloha = *Onc*. Goldiana × *Onc*. Star Wars. ^3^*Mtssa *Charles = *Brassia verrucosa *× *Milt. spectabilis*.

Fifteen commercial Oncidiinae varieties were obtained from a grower (Yung Hsin Orchid nursery) in Taichung, Taiwan, including four *Oncidium *(*Onc*. Gower Ramsey, Gower Ramsey 'Lemon heart', Gower Ramsey 'Sunkiss', and Sweet Sugar 'Million Coins'), five *Beallara *(*Bllra*. Eurostar, Peggy Ruth Carpenter 'Morning Joy', Marfitch 'Howard Dream', Tahoma Glacier 'Sugar Sweet' and Smile Eri), two *Odontoglossum *(*Odm*. Margarete Holm and Violetta von Holm), two *Odontocidium *(*Odcdm*. Golden Gate, *Odcdm*. Wildcat 'Garfield'), one *Degarmoara *(*Dgmra*. Flying High) and one *Zelenkocidium *(*Zelenkocidium *Little Angel). These orchids were maintained in the greenhouse at Academia Sinica, Taipei, Taiwan, and vouchers specimens were deposited at the National Natural and Science Museum, Taichung, Taiwan. Leaves from these orchids were used in this study. Details of the parents of these species are shown in Figure 1.

### DNA purification, primer design and genomic PCR

The PCR strategy for sequencing the chloroplast genome was adapted from Wu et al. [7]. For the chloroplast genomic PCR analysis, total genomic DNA from greenhouse-grown plants was isolated using a urea extraction buffer system [11]. The coding regions of the *P. aphrodite *chloroplast genome were used as the templates for primer design. A series of overlapping DNA fragments of 2 to 3 kb were amplified using specific primers (Additional file 1). The overlaps between adjacent PCR fragments were about 200 bp. The PCR amplification program consisted of 30 cycles of at 94°C for 30 s, at 55°C for 30 s and at 72°C for 90 s. The PCR products were sequenced. DNA sequencing was carried out with the Big-Dye Terminator Cycle Sequencing kit using an ABI Prism 3,700 DNA analyzer (Applied Biosystems, Foster City, CA). All gaps were filled by designing new primers on the basis of sequences obtained from PCR products (Additional file 1). The sequences were verified by comparison with the chloroplast genome of *P. aphrodite *using the VectorNTI AlignX software program (vers. 7.0; Invitrogen, Carlsbad, CA; parameters: overlap: 30; identity: 0.95; cutoff score: 40).

### Broad Phylogenetic analysis

Analyses of 48 species were performed using the same 61 conserved protein-coding genes analyzed in previous studies [12-15]. This set of loci was assembled from the aligned Nexus file for 45 species that is supplemental to the paper by Hansen et al. [[14]; available from http://chloroplast.cbio.psu.edu/organism.cgi]. Also included were sequences from *Lemna minor *(GenBank accession NC_010109), *Joinvillea plicata *(GeneBank accessions FJ486219 - FJ486269, L01471, U21973, and AF001864), and *Hordeum vulgare *(NC_008590) to increase sampling among monocots and break up putative long branches. Gaps introduced by the alignment were excluded from phylogenetic analyses. Two phylogenetic methods were used-- maximum likelihood (ML), implemented in GARLI vers. 0.951-1 [16], and maximum parsimony (MP), implemented in PAUP* vers. 4.0b10 [17]. ML analyses were run under the general time reversible model, with all parameters estimated. A heuristic search of 100 random addition replicates was conducted for the MP analyses. Nonparametric bootstrap analyses were also performed with 100 (ML) or 1000 (MP) pseudoreplicates [18]. *Ginkgo biloba *was the specified outgroup for all analyses [14].

### Contig assembly and annotation

VectorNTI Contig Express was used to assemble contigs (parameters: overlap: 30; identity: 0.95; and cutoff score: 40). The chloroplast genome was annotated using DOGMA (Dual Organellar GenoMe Annotator) [19]. This program uses a FASTA-formatted input file of the complete genomic sequences and identifies putative protein-coding genes by performing BLASTX searches against a custom database of published chloroplast genomes. Both tRNAs and rRNAs were identified by BLASTN searches against the same database of chloroplast genomes. For genes with low sequence identity, manual annotation was performed after identifying the position of the start and stop codons, as well as the translated amino acid sequence, using the chloroplast/bacterial genetic code.

### Analysis of variability in *ndh *genes of 15 *Oncidium *varieties

**Figure 2:**
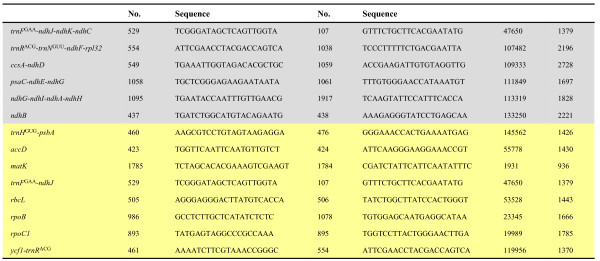
**Primers for Oncidiinae *ndh *gene and phylogenetic analysis**. ^1^Primer sequences, annealing site of the forward primer in *Onc*. Gower Ramsey and the anticipated amplicon size (bp) are presented. ^2^Different background colors indicate different experiments; gray: *ndh *gene identification; yellow: phylogenetic analysis.

To investigate the *ndh *genes of Oncidiinae, six cpDNA regions (*trnF*^GAA^-*ndhJ-ndhK-ndhC*, *trnR*^ACG^-*trnN*^GUU^-*ndhF-rpl32*, *ccsA-ndhD*, *psaC-ndhE-ndhG*, *ndhG-ndhI-ndhA-ndhH *and *ndhB*) were obtained by a PCR approach from the 15 varieties as indicated in Methods (Accession no.: GU175359-GU175415, Additional file 2). The primer sequences, sequence size and sequence position in *Onc*. Gower Ramsey of these regions in *Onc*. Gower Ramsey are shown in Figure 2.

### Phylogenetic analysis of 15 *Oncidium *varieties

To investigate the phylogenetic relationships between Oncidiinae at the species level, eight cpDNA regions [intergene region (*trnH*^GUG^-*psbA*, *trnF*^GAA^-*ndhJ*, *ycf1*-*trnR*^ACG^) and coding regions (*accD*, *matK*, *rbcL*, *rpoB*, and *rpoC1*)] were obtained by PCR from plastid DNA of the leaves of the 15 varieties as above (Accession no.: GQ915119-GQ915133; GU132947-132992; GU136249-GU136275; GU175340-GU175358, Additional file 2). The primer sequences, sequence size and sequence position of these regions in *Onc*. Gower Ramsey are shown in Figure 2. Phylogenetics were conducted using MEGA4 (gap opening penalty: 15; gap extension penalty: 6.66; DNA weight matrix: IUB; transition weight: 0.5; negative matrix: off; and delay divergent cutoff: 30%) [20]. The evolutionary history was inferred using the maximum parsimony, minimum evolution (ME), neighbor-joining (NJ) and unweighted pair-group method with arithmetic mean methods. In these four analyses, the bootstrap consensus tree was inferred from 1000 replicates [18]. Branches corresponding to partitions reproduced in < 50% bootstrap replicates were collapsed. The values of replicate trees in which the associated taxa clustered together in the bootstrap test (1000 replicates) are shown next to the branches [18].

## Results

### *Oncidium *chloroplast genome sequencing

**Figure 3:**
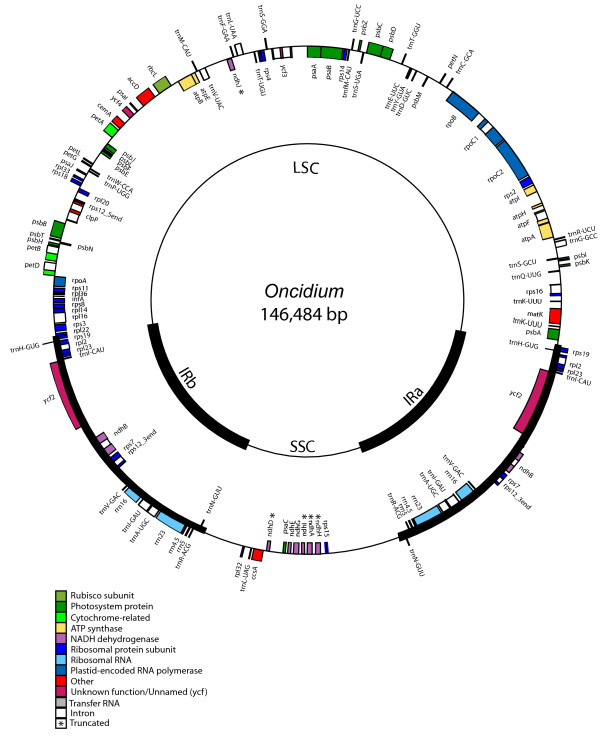
**Gene map of *Onc*. Gower Ramsey chloroplast genome**. The thick lines indicate the extent of the IRa and IRb, which separate the genome into SSC and LSC regions. Genes on the outside of the map are transcribed clockwise and genes on the inside of the map are transcribed counterclockwise.

The size of the *Onc*. Gower Ramsey chloroplast genome is 146,484 bp (Figure 3). The genome includes a pair of IRs of 25,755 bp each, a SSC region of 12,650 bp, and a LSC region of 82,324 bp. The *Onc*. Gower Ramsey chloroplast genome contains 101 different genes, of which 16 are duplicated in the IR, giving a total of 133 genes. There are 29 distinct tRNAs, six of which are duplicated in the IR. Sixteen genes contain one or two introns, with six of the introns in tRNAs. Coding regions make up 49.94% of the chloroplast genome (41.86% protein-coding genes, 8.08% RNA genes) and non-coding regions, which contain intergenic spacer (IGS) regions and introns, comprise 50.06%. The overall GC and AT content of the chloroplast genome is 37.32% and 62.68%, respectively. The gene order of *Onc*. Gower Ramsey cpDNA is similar to that of the orchid *P. aphrodite *(Figure 3). The *rps15 *gene is not included in the IR. In contrast with the chloroplast genomes of Poaceae, *Onc*. Gower Ramsey contained introns in the *clpP and rpoC1 *loci and had intact copies of the *accD*, and *ycf2 *genes, which are incomplete or entirely missing in Poaceae.

**Figure 4:**
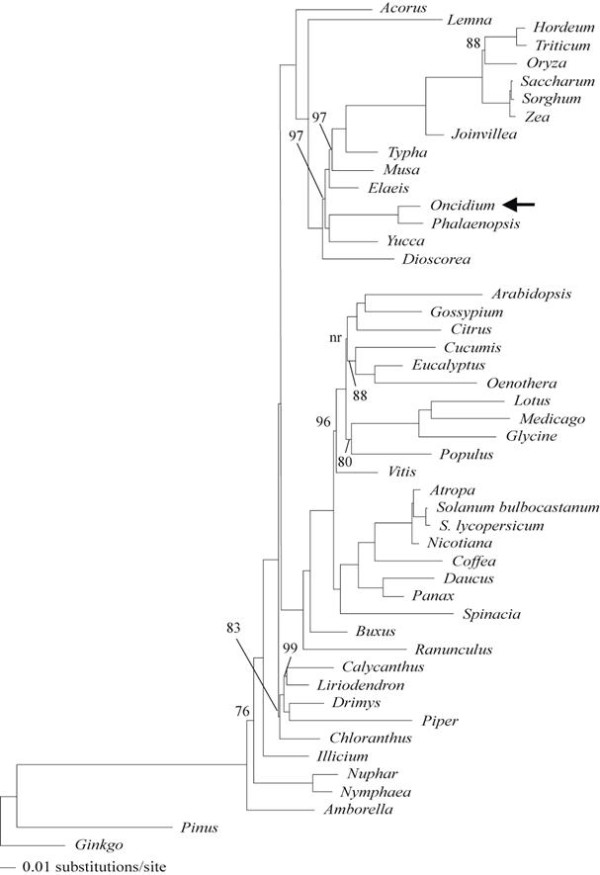
**Maximum likelihood phylogram for 61 conserved protein-coding genes**. All nodes have 100% ML bootstrap support unless otherwise indicated. Horizontal branch lengths are proportional to the number of inferred substitutions/site along that branch. One node, marked "nr," was not resolved in the ML bootstrap consensus tree. The position of *Oncidium *in a clade of Asparagales is indicated with an arrow.

The broad phylogenetic analysis resulted in two trees, an ML tree with -lnL = 412281.26 (Figure 4) and an MP tree of 75,521 steps and 14,974 parsimony informative characters. The MP tree had a a consistency index (excluding uninformative characters) of 0.3649 and a retention index of 0.5997 (tree not shown). The topologies of the monocot subtrees were identical for the two analyses in which *Oncidium *was maximally supported as the sister of *Phalaenopsis *and the two orchids were united with *Yucca*, another representative of Asparagales, with maximum support.

### Analysis of variability in *ndh *genes of 15 *Oncidium *species

**Figure 5:**
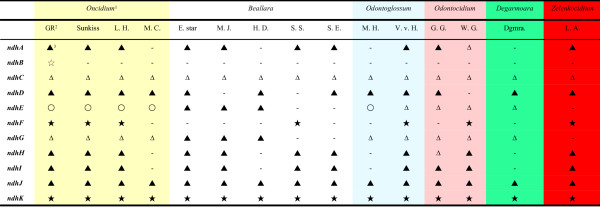
**Summary of *ndh *gene patterns in Oncidiinae**. ^1^Different background colors indicate different genera; yellow: *Oncidium*, white: *Beallara*, blue: *Odontoglossum*, pink: *Odontocidium*, purple: *Colmanara*, green: *Degarmoara*, red:*Zelenkocidium*. ^2^GR: Gower Ramsey, Sunkiss: Gower Ramsey 'Sunkiss', L. H.: Lemon heart, M. C.: Sweet sugar 'Million Coin', E. star: Eurostar, M. J.: Peggy Ruth Carpenter 'Morning Joy', H. D.: Marfitch 'Howard Dream', S. S.: Tahoma Glacier 'Sugar Sweet', S. E.: Smile Eri, M. H.: Margarete Holm, V. v. H.: Violetta. von Holm, G.G.: Golden Gate, W.G.: Wildcat 'Garfield', Dgmra: *Dgmra*. Flying High, L. A.: Little Angel. ^3^'black star': absent genes (no sequence exists). 'white star': stop codon (There is no change in gene size but there are stop codons within coding sequences). 'black triangle': truncated genes (only partial coding sequences are observed). 'white triangle': frame shift (reading frame shifted or nucleotides deleted). 'white circle': functional protein., -: no PCR product obtained using the primers in Figure 2.

**Figure 6:**
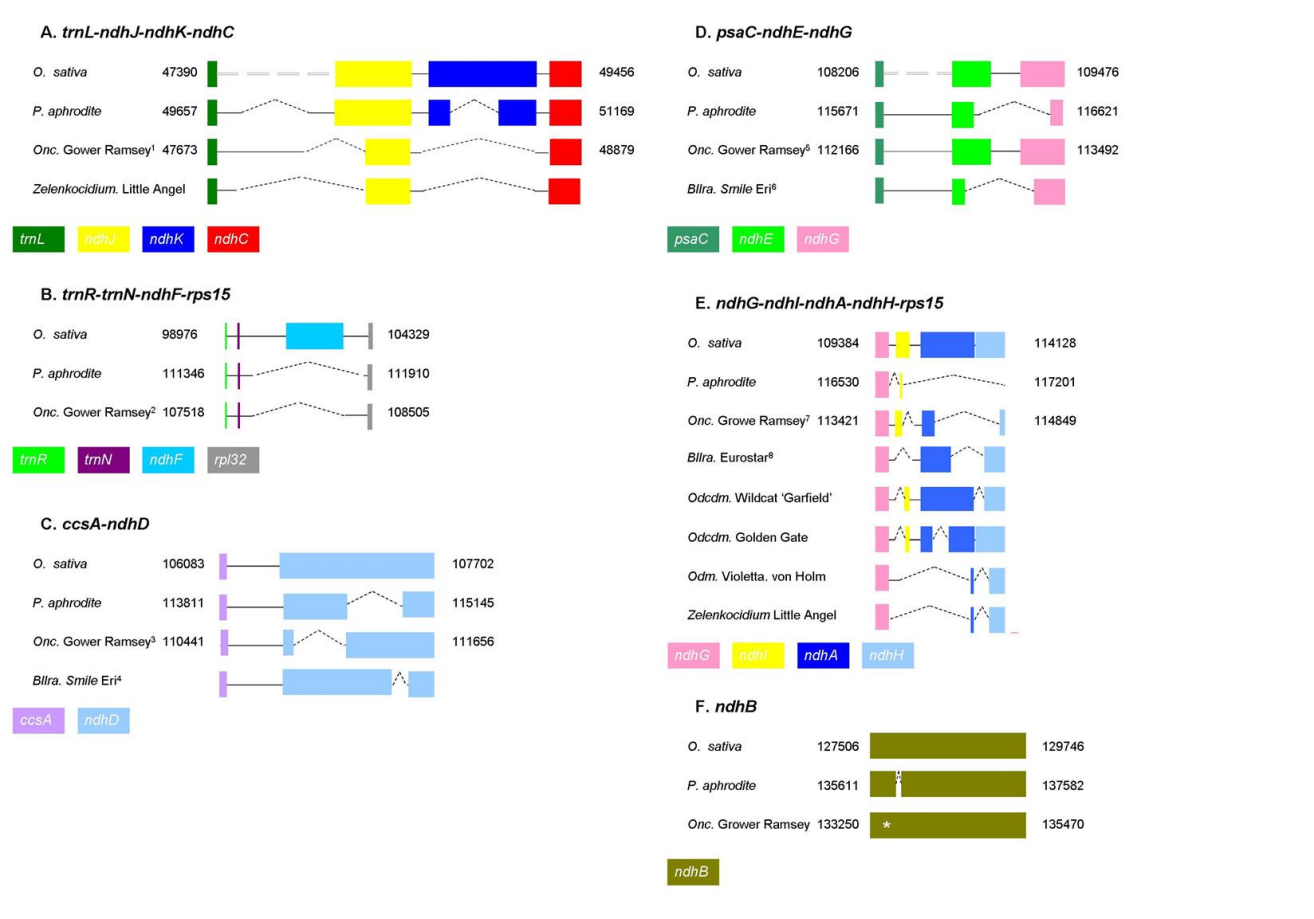
**Structure of *ndh *genes are different in Oncidiinae varieties**. Numbers indicate the positions in the chloroplast genome. The angled dashed lines indicate the gaps. Different colors indicate different *ndh *genes, a color key is shown at the bottom of each part of the Figure. Detailed information is shown in Figure 5. ^1 ^including most of the Oncidiinae except *Zelenkocidium *Little Angel. ^2^including three *Oncidium *Gower Ramsey varieties, *Bllra*. Tahoma Glacier 'Sugar Sweet', *Odm*. Violetta, von Holm, *Odcdm*. Wild cat 'Garfield', and *Zelenkoncidium *Little Angel. ^3^including four *Oncidium *varieties, two *Odontoglossum *varieties, one *Odontocidium*, *Dgmra *Fly High, and *Zelenkoncidium *Little Angel. ^4 ^including *Bllra*. Eurostar, *Bllra*. Marfitch 'Howard Dream', and *Bllra*. Smile Eri. ^5^including four *Oncidium *varieties, two *Odontoglossum *varieties, two *Odontocidium *varieties, *Dgmra*. Fly High. ^6^including *Bllra*. Eurostar, *Bllra*. Peggy Ruth Carpenter, and *Bllra*. Marfitch 'Howard Dream'. ^7^including three *Oncidium *varieties. ^8^including *Bllra*. Eurostar, *Bllra*. Tahoma Glacier 'Sugar Sweet', *Bllra *Peggy Ruth Carpenter and *Bllra*. Smile Eri.

Six cp DNA regions (*trnF*^GAA^-*ndhJ-ndhK-ndhC*, *trnR*^ACG^-*trnN*^GUU^-*ndhF-rpl32*, *ccsA-ndhD*, *psaC-ndhE-ndhG*, and *ndhG-ndhI-ndhA-ndhH *and *ndhB*) were obtained by PCR from total DNA of the leaves of the 15 varieties. Most of the *ndh *genes in the 15 Oncidiinae varieties, with the exception of *ndhE *in some species, had no function (Figure 5). In all 15 of the Oncidiinae varieties studied, the *ndhJ *gene was truncated (partial sequence remained) and the *ndhK *gene was absent (no sequence exists). In *ndhC*, a frame shift occurred, creating a stop codon in the middle of the gene in all Oncidiinae, including *Onc*. Gower Ramsey, resulting from a 17 bp deletion (Figure 6A, Figure 5).

The *ndhB *of Oncidiinae does not function due to a stop codon in the first exon. To date, six orchid *ndhB *genes, including *P. aphrodite*, have been cloned and published in the NCBI database. Two of them could translate putative functional *ndhB *protein [*Orchis rotundifolia *(Accession no.: AY147484) and *Coelogyne crisata *(Accession no.: AY147475)]. There is also a frame shift in the second exon of *Cypripedium passerinum *(Accession no.: AY147479, AY147478.1). That of *Odontoglossum crispum *(AY834278) is only a partial sequence that could translate a putative *ndhB *protein.

The *ndhF *locus, which is located in the LSC-IRa junction, was absent in the Oncidiinae varieties. Notably, the nucleotide deletions in the *trnR*^ACG^-*trnN*^GUU^-*ndhF-rpl32 *region were different between *P. aphrodite *and Oncidiinae (Figure 6B, Figure 5).

All of the 12 Oncidiinae *ndhD *genes cloned here were truncated. The overall pattern of truncation can be classified into two types: a truncation occurring at the 3'-end of *ndhD *(as in the *Bllra*. varieties) and a truncation in the 5'-end (in the rest of the clones tested) (Figure 6C, Figure 5).

The sequences of *Onc*. Gower Ramsey varieties, *Onc*. Sweet Sugar and *Odm*. Margarete Holm indicate that the translation capacity of *ndhE *is retained in these species (Figure 5). Of the species with modified *ndhE *genes, *Odm*. Violetta. von Holm, *Odcdm*. Golden Gate and *Odcdm*. Wildcat contained frame shifts; *Dgmra*. Flying High had a 30 bp deletion; and there were deletions of over 30 bp in the four *Beallara *varieties (Figure 6D, Figure 5).

Although nine varieties had no deletions in the *ndhG *genes, these varieties had three internal stop codons within *ndhG*, rendering *ndhG *inactive (Figure 5). There was a deletion of about 250 bp in the *ndhG *gene of the *Beallara *species (Figure 6D, Figure 5).

The region encompassing *ndhI *is the most complicated of the chloroplast *ndh *gene regions. Generally, genes from the same genus had the same pattern (for example, see *Oncidium *and *Beallara*, Figure 6E, Figure 5). In the *Onc*. Gower Ramsey varieties, the *ndhI *gene was partially deleted, and in the *Beallara *varieties, *Zelenkocidium *Little Angel and *Odm*. Violetta. von Holm, the *ndhI *gene was completely absent.

Truncated *ndhA-ndhH *genes still existed in most of the Oncidiinae species in this study. With the exception of frame shifts in *Oncdm*. Garfield *ndhA *and *Oncdm*. Golden Gate *ndhH*, the other *ndhA *through *ndhH *genes in the other five genera all showed deletions of various types.

### Phylogenetic analysis of 15 *Oncidium *species

Based on the amount of variation in the cpDNA and congruence with parent relationships, certain chloroplast regions were determined to be more useful than others. Because *rbcL *is highly conserved; bootstrap scores are lower then 50% and are not useful for determining parent relationship (Additional file 3). Using the *accD gene*, only the species belonging to *Beallara *and *Oncidium *could be separated as the pattern and relationships among other species were not correlated with the parent relationship (Additional file 4).

**Figure 7:**
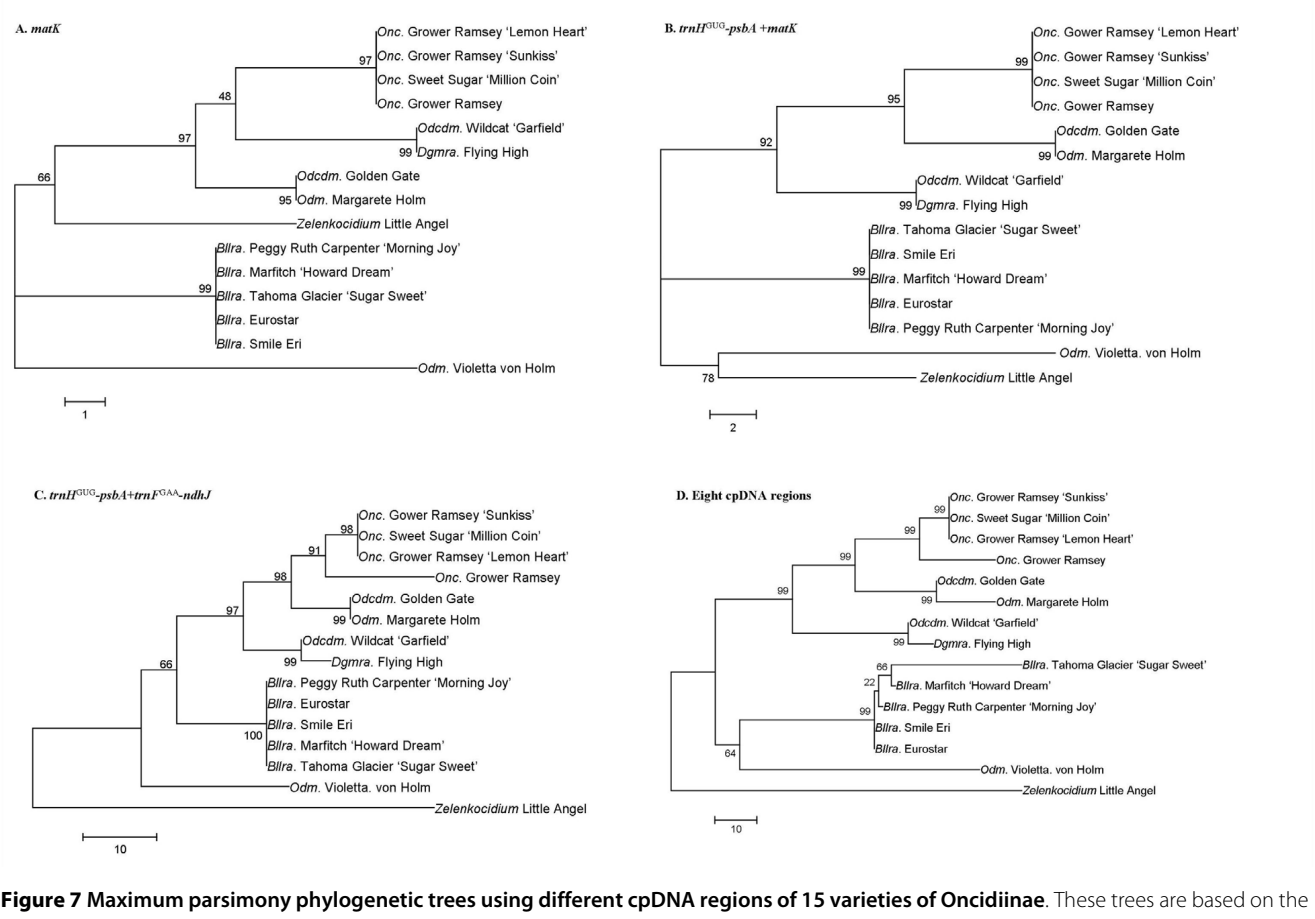
**Maximum parsimony phylogenetic trees using different cpDNA regions of 15 varieties of Oncidiinae**. These trees are based on the nucleotide sequences of (A) *matK *(B) *trnH*^GUG^-*psbA*+*matK *(C) *trnH*^GUG^-*psbA*+*trnF*^GAA^-*ndhJ *(D) from all eight cpDNA regions. The numbers at the nodes indicate bootstrap support values. The scale bar indicates a branch length corresponding to 100 character-state changes.

In the *matK *region, the phylogenetic analysis of these sequences and 15 economic varieties gave results that correlated with parent relationship (Figure 7A). Therefore, we combined the most diverse cpDNA regions, *trnH -psbA *[21], *matK *and *trnF*^GAA^-*ndhJ *[22], for phylogenetic analysis. The *trnH*^GUG^-*psbA *and *ndhJ *combination provided the most similar results to those obtained from all eight cpDNA regions (Figure 7).

## Discussion

### Using PCR to sequencing Oncidium Gower Ramsey and phylogenomic applications

Although there are many methods for cp genome sequencing, PCR is one method that is easy and economical [7]. However, the gene content and order in monocot cp genomes is relatively diverse so that the use of the *P. aphrodite *as a template for primer design in this study was limiting. This was especially true in the *ndh *gene regions where the deletion of *ndh *genes in *Oncidium *is very different from that in *P. aphrodite*. Furthermore, when using PCR methods with total genomic DNA as the template, some of the cp sequence regions are similar to those in other organelles, thus raising the possibility of false results. To prevent such results, we used BLAST analysis and different combinations of primers to amplify the same region.

Considerable effort is being expended to investigate phylogenomic relationships among monocots using cp genomes (see http://www.botany.wisc.edu/monatol/). Here, the phylogenetic position of Orchidaceae among Asparagales is confirmed with the robust support provided by many informative cpDNA characters. Further sampling among orchids in the future phylogenomic studies building on our results will clarify the complex relationships within the large family. Therefore, the cpDNA of *Oncidium *Gower Ramsey provides valuable information for further orchid cp genome sequencing and phylogenomics.

### *ndh *genes in Oncidiinae cpDNA

In higher plant chloroplasts, the NAD(P)H dehydrogenase (NDH) complex functions in PSI cyclic electron flow and chlororespiration [23]. Eleven subunits of the chloroplast *ndh *genes (*ndhA*-*ndhK*) are encoded in the chloroplast genome. In addition 3 cyanobacterial orthologs, nuclear-encoded subunits genes (*NdhM*-*NdhO*), have also been identified in chloroplasts [24]. This indicates that nucleus-encoded *ndh *genes originated in cyanobacteria and were transferred from the chloroplast genome to the nuclear genome during evolution [25]. However, in *Onc*. Gower Ramsey, out of all the 11 chloroplast-encoded *ndh *genes, only *ndhE *theoretically translates into a functional protein. This *ndh *gene truncation and absence was also observed in *P. aphrodite *[8]. Using a PCR approach to sequence the *ndh *genes of 15 varieties, we demonstrated that truncation and absence of *ndh *genes from the cp is a general phenomenon in Oncidiinae.

The loss-of-function of *ndh *genes or other chloroplast-encoded genes occurs in many plants, such as parasitic plants [26-30] and achlorophyllous orchids [31,32]. Loss-of-function in *ndh *genes occurs not only in heterotrophic plants, but also in autotrophic species. In *Pinus thunbergii*, all 11 *ndh *genes were putative loss-of-function alleles [33], and in another Coniferales species, *Keteleeria davidiana*, was also found to contain nonfunctional *ndh *genes [34]. In three Gnetophytes, which comprise three related families of woody gymnosperms (*Welwitschia mirabilis*, *Ephedra equisetina*, and *Gnetum parvifolium*), all 11 *ndh *genes are non-functional, 10 being absent and one, *ndhB*, being a pseudogene [34,35]. It is interesting to note that this *ndh *deletion does not occur in all gymnosperm species. The *ndh *genes exist in the chloroplast genomes of *Cryptomeria *and *Cycas *[36,37]. It is possible that ancestral plastid *ndh *genes were transferred to the nucleus, remaining functional to this day [8,33].

Loss-of-function *ndh *genes also occur in other orchids [38]. *Phaelenopsis aphrodite *lacks the *ndhA*, *ndhF*, and *ndhH *genes, and only remnants of the other eight subunits sequences were found [8]. The 11 *ndh *genes were either truncated or frame-shifted, suggesting that they are nonfunctional [8]. In this report, we demonstrated that *ndh *gene deletion is also common in Oncidiinae: the deletion pattern differs not only between *Oncidium *and *Phalaenopsis *(Figure 6), but even within the 15 Oncidiinae species analyzed (Figure 5).

From a physiological view, since parasitic plants obtain organic nutrients from the host, loss of functional *ndh *genes from the chloroplast is not surprising. However, this does not explain why most *ndh *genes are non-functional or deleted in autotrophic plants. The presence of *ndh *homologs encoded within the nucleus was confirmed using PCR assays of total DNA of *Phalaenopsis *[8]. The resulting sequences are in frame and imply that the ancestral functional *ndh *copies of the plastid genome may have been transferred to the nuclear genome [8].

### Phylogenetic analysis of 15 *Oncidium *species

Because it is easy to perform interspecific or intergeneric crosses with orchids, there are many artificial intergeneric hybrids. These hybrids are not distinct phenotypically and are partially named according to their parental background. However, hybrids with different parental backgrounds may be classified into the same genus. In addition, Hybrids from differently named genera may originate from the same female parent. Economic varieties of orchids are generally hybrids of other hybrids and some of the parental information has been lost. To further complicate matters, changes in the names of genera and taxonomy of the Oncidiinae are frequent. In 2004, the names of more than 2200 hybrids comprising some 20% of the *Oncidium *group were changed. For example, *Colmanara *Wildcat was changed to *Odcdm*. Wildcat and *Oncidium *Little Angel was changed to *Zelenkocidium *Little Angel. These changes and whether there were grounds for them could be clarified by looking carefully at the cpDNA, which could identify the female parent.

Among the eight sequences studied here, the phylogenetic analysis using *matK *was most well-correlated with the parent relationship (Figure 7A). There are at least three advantages of using the *matK *region for phylogenetic analysis: (1) this region is variable at the interspecies level [22]; (2) this region is easy to amplify using published primer sequences [39]; and (3) a large amount of sequence information about Oncidiinae *matK *is readily available in the public domain, including the number of sequences (695) and the length of the sequences (791 bp). Here, we performed a phylogenetic analysis by using 15 varieties and their 180 related sequences. Among the results we found several areas of divergence between the taxonomy of Oncidiinae based on morphology and our phylogenetic analyses. For example, the female parent of *Beallara *is *Miltassia*, making the grandparent *Brassia*. The sequences of *Beallara *were highly correlated with other *Brassia *species, and most closely with the female parent *Brassia verrucosa *(Accession no.: EF079203, data not shown). However, the phylogenetic analysis of these sequences showed that the *Odontoglossum matK *was dispersed around the *Oncidium *group (data no shown). Result such as these suggests that analysis of a single region may not contain enough information for interspecies phylogenetic analysis.

To solve this problem, available sequence information must be increased. During phylogenetic analysis, correlation is dependent on the length and properties of DNA or amino acid information. Because the information on orchid cpDNA is limited, the combination of several sequences derived by PCR using universal primers could be a successful strategy [see [22,31,32,40,41]]. In this report, eight sequences from each species were combined (total length of 7042 bp) and were well-correlated with the parent relationship. However, to manage labor and supply costs, we wanted to identify the smallest region that would result in the same performance as using all eight regions. Therefore, we combined divergent cpDNA sequences such as *matK *for further analyses. In addition to *matK*, the *trnH*^GUG^-*psbA *region is another divergent cpDNA region useful for phylogenetic analysis [21,22]. Various expansions or contractions of inverted repeats (IRs) in chloroplast genomes lead to diverse *trnH*^GUG^-*psbA *regions [42-44]. The structural changes in cpDNA provide useful phylogenetic inferences [45]. According to these data, the *trnH*^GUG^-*psbA *regions are information-rich and could be used for phylogenetic analysis.

In addition, *ndh *gene deletion is a unique feature that may also provide useful information for parentage analysis. The *trnF*^GAA^-*ndhJ-ndhK-ndhC *region could be amplified by PCR in all of the 15 varieties. Therefore, different combinations of these information-rich regions (*matK*, *trnH*^GUG^-*psbA *and *trnF*^GAA^-*ndhJ*) were used for phylogenetic analysis. According to our results, two variable cpDNA regions, *trnH*^GUG^-*psbA *and *trnF*^GAA^-*ndhJ*, could provide sufficient information for genus-to-species level phylogenetic analysis.

However, several questions require further investigation. The first is the placement of *Odm*. Violetta von Holm, whose female parent is *Odm. bictoniense*. Irrespective of the cpDNA template, *Odm*. Violetta von Holm did not correlate with *Odcdm*. Golden Gate or *Odm*. Margarete Holm, which are both derived from the same female parent. The second is the placement of *Dgmra*. Flying High, which has the female parent *Mtssa*. Jet Setter. Theoretically, the cpDNA of *Dgmra*. Flying High should be closely related to *Beallara *species, which are derived from a *Miltassia *female parent; however our data indicated that *Dgmra*. Flying High is more similar to *Odontoglossum*.

There are many advantages to using cpDNA for phylogenetic and parentage analysis. But this genetic information is only derived from the female parent. Therefore, in the future nuclear genes also need to be analyzed for parentage analysis [46]. In *Pleione*, the nrITS region was found to be more variable than the plastid regions sequenced, and nrITS gene trees were largely congruent with those inferred from the plastid regions [46]. Our data here suggest that the taxonomy of the Oncidiinae may be improved by both chloroplast and nuclear genome analysis.

## Conclusion

In this report, we used *P. aphrodite *as a prototype to design primers to complete the *Onc*. Gower Ramsey genome sequence. The primers and the genome sequence information obtained will be useful for further orchid cpDNA sequencing and broad phylogenetic analyses among monocots. Gene annotation showed that most of the *ndh *genes in Oncidiinae are non-functional, with the exception of *ndhE*, which could theoretically produce a functional protein. In the previous reports, non-functionality of *ndh *genes has been found in photosynthetic orchids and gymnosperms, such as in *Pinus thunbergii *and *Phalaenopsis*. In this report, using a PCR approach, we identified the *ndh *genes in different Oncidiinae plants. The *ndh *genes were also non-functional in most of the plants tested, except for *ndhE *in four *Oncidium *species and *Odm*. Margarete Holm. These genes would be useful for parentage analysis. The non-protein coding regions *trnH*^GUG^-*psbA *and *trnF*^GAA^-*ndhJ *were also determined to be cpDNA regions that would be the most useful for phylogenetic analysis. When these regions were checked in commercial varieties, most confirmed to previously known inheritance information; however, some variations need further investigation. Also, to confirm and complement the results obtained from cpDNA, genetic information may also be derived from nuclear DNA. We conclude that complete chloroplast genome information is useful for plant phylogenetic and evolutionary studies in *Oncidium *breeding and variety identification.

## Competing interests

The authors declare that they have no competing interests.

## Authors' contributions

FHW, MTC, DCL, and CTH performed PCR and primer design. YWL performed the bioinformatic analysis. HD contributed to chloroplast genome annotation, correcting errors in genome sequence, assembling the genome map and participated in manuscript preparation. MRD performed broad phylogenetic analyses and participated in manuscript preparation. CSL conceived this project, supervised PCR, primer design, bioinformatic analysis, and participated in the preparation of the manuscript. All authors read and approved the final manuscript.

## Supplementary Material

Additional file 1 **Primers for *Oncidium *Gower Ramsey chloroplast sequencing**. Excel file containing Primers for *Oncidium *Gower Ramsey chloroplast sequencing. Click here for file

Additional file 2 **The accession numbers of the Oncidiinae chloroplast sequences for *ndh *gene and phylogenetic analysis**. Excel file containing the accession numbers of the Oncidiinae chloroplast sequences for *ndh *gene and phylogenetic analysis. Click here for file

Additional file 3 **Maximum parsimony phylogenetic trees using *rbcL *regions of 15 varieties of Oncidiinae**. These trees are based on the nucleotide sequences of *rbcL *sequences. The numbers at the nodes indicate bootstrap support values. The scale bar indicates a branch length corresponding to 100 character-state changes. Click here for file

Additional file 4 **Maximum parsimony phylogenetic trees using *accD *regions of 15 varieties of Oncidiinae**. These trees are based on the nucleotide sequences of *accD *sequences. The numbers at the nodes indicate bootstrap support values. The scale bar indicates a branch length corresponding to 100 character-state changes. Click here for file
