# Supplementary figures and images for: Complete chloroplast genome of Oncidium Gower Ramsey and evaluation of molecular markers for identification and breeding in Oncidiinae
Source: BMC Plant Biol. 2010 Apr 16;10:68. doi: 10.1186/1471-2229-10-68 (PMC3095342; doi:10.1186/1471-2229-10-68)

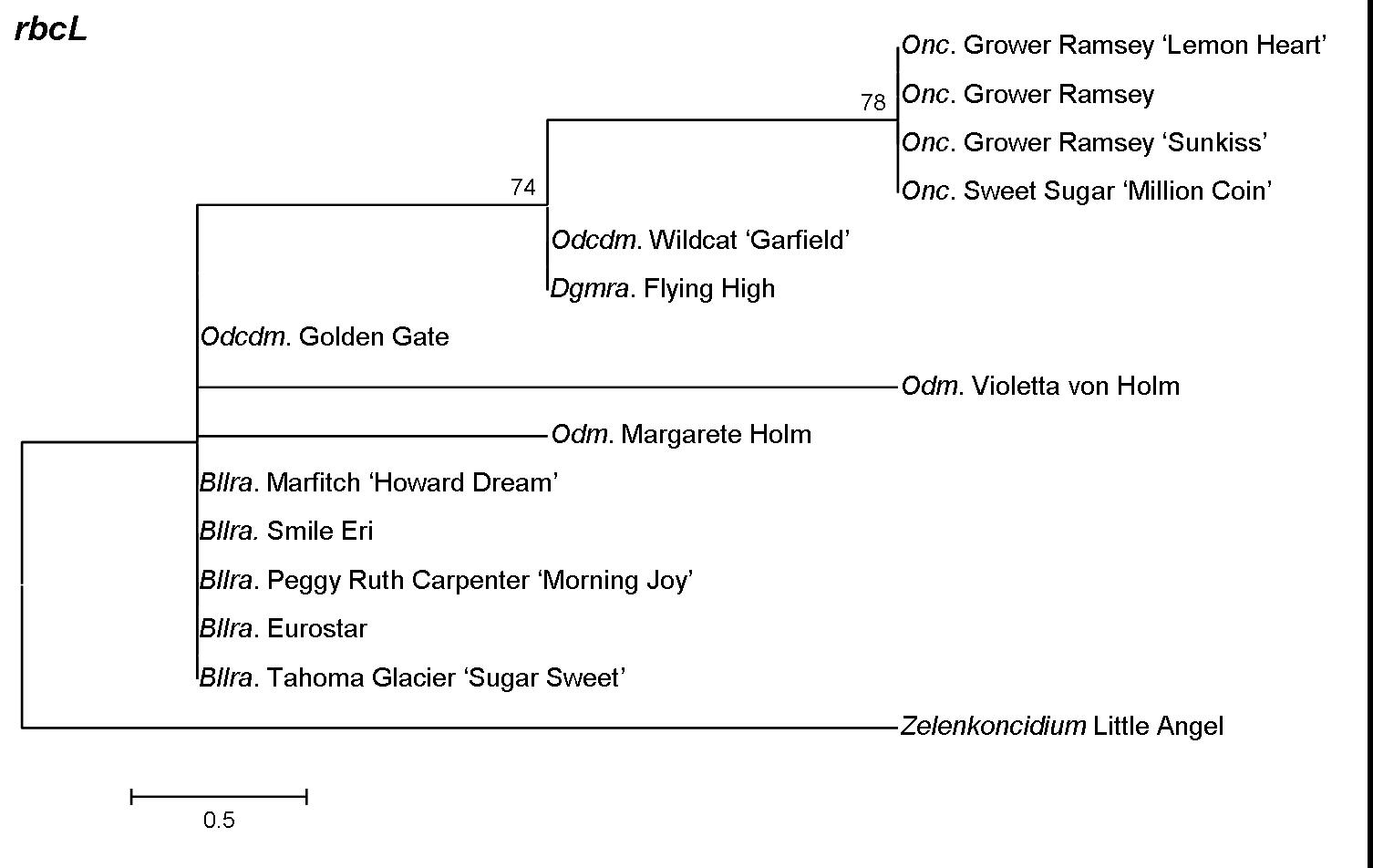

Supplement: Additional file 3 — Maximum parsimony phylogenetic trees using rbcL regions of 15 varieties of Oncidiinae. These trees are based on the nucleotide sequences of rbcL sequences. The numbers at the nodes indicate bootstrap support values. The scale bar indicates a branch length corresponding to 100 character-state changes. [file 1471-2229-10-68-S3.JPEG]

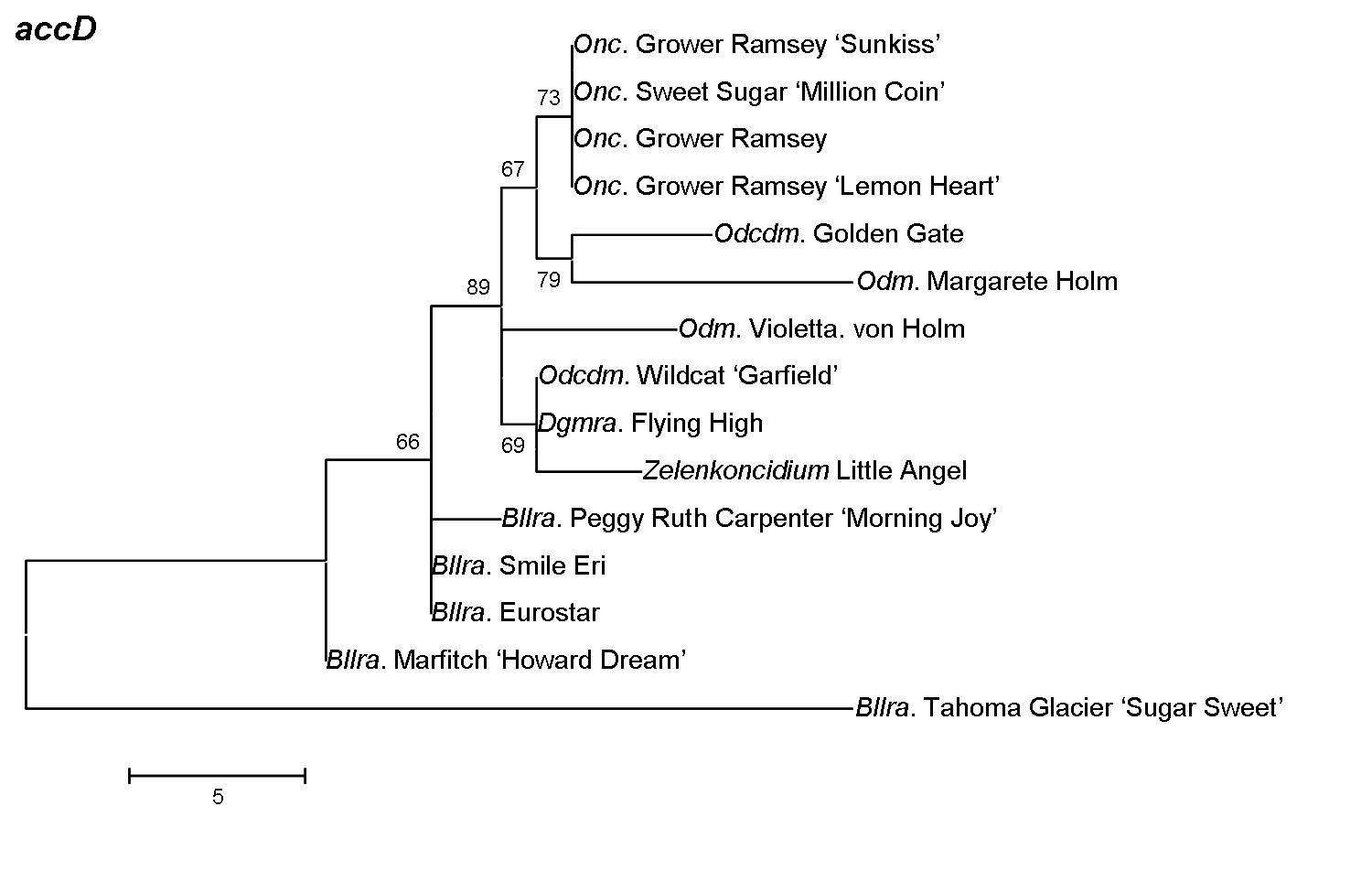

Supplement: Additional file 4 — Maximum parsimony phylogenetic trees using accD regions of 15 varieties of Oncidiinae. These trees are based on the nucleotide sequences of accD sequences. The numbers at the nodes indicate bootstrap support values. The scale bar indicates a branch length corresponding to 100 character-state changes. [file 1471-2229-10-68-S4.JPEG]
